# Genetic mapping and functional analysis of a classical tassel branch number mutant *Tp2* in maize

**DOI:** 10.3389/fpls.2023.1183697

**Published:** 2023-06-02

**Authors:** Juan Li, Xi Wang, Junfeng Wei, Xinxin Miao, Xiaoyang Shang, Lin Li

**Affiliations:** ^1^ National Key Laboratory of Crop Genetic Improvement, Huazhong Agricultural University, Wuhan, China; ^2^ Hubei Hongshan Laboratory, Wuhan, China

**Keywords:** *Teopod2*, tassel branch number, genetic mapping, functional analysis, *zma-miR156h*

## Abstract

Tassel branch number is a key trait that contributes greatly to grain yield in maize (*Zea mays*). We obtained a classical mutant from maize genetics cooperation stock center, *Teopod2* (*Tp2*), which exhibits severely decreased tassel branch. We conducted a comprehensive study, including phenotypic investigation, genetic mapping, transcriptome analysis, overexpression and CRISPR knock-out, and tsCUT&Tag of *Tp2* gene for the molecular dissection of *Tp2* mutant. Phenotypic investigation showed that it is a pleiotropic dominant mutant, which is mapped to an interval of approximately 139-kb on Chromosome 10 harboring two genes *Zm00001d025786* and *zma-miR156h*. Transcriptome analysis showed that the relative expression level of *zma-miR156h* was significantly increased in mutants. Meanwhile, overexpression of *zma-miR156h* and knockout materials of *ZmSBP13* exhibited significantly decreased tassel branch number, a similar phenotype with *Tp2* mutant, suggesting that *zma-miR156h* is the causal gene of *Tp2* and targets *ZmSBP13* gene. Besides, the potential downstream genes of *ZmSBP13* were uncovered and showed that it may target multiple proteins to regulate inflorescence structure. Overall, we characterized and cloned *Tp2* mutant, and proposed a zma-miR156h-ZmSBP13 model functioning in regulating tassel branch development in maize, which is an essential measure to satisfy the increasing demands of cereals.

## 1 Introduction

Maize (*Zea mays*) is a staple crop that is experiencing a steady increase in both planting area and crop yields due to the rising population, diet shifts, and increasing biofuel consumption (Tilman et al., 2011). However, to ensure food security, increasing crop yields through sustainable means is more viable than expanding the land used for food production (Godfray et al., 2010; Phalan et al., 2011). Consequently, breeders are continually selecting cultivars that own optimal plant architecture to increase maize grain yield while minimizing land usage. To this end, breeders currently focus on studying morphological traits such as leaf, root, stem, and inflorescence morphology, which constitute maize plant architecture, for selective breeding. By doing so, they aim to identify and select ideal maize plant architecture that will maximize yield.

The Poaceae family is one of the largest angiosperm families and has evolved complex inflorescence morphologies, such as branches and spikelets (Zhang and Yuan, 2014). In maize, the inflorescence structures initially derive from inflorescence meristem (IM) which is responsible for initiating lateral primordia (Wang et al., 2022). IM is transferred from shoot apical meristem (SAM), which initiates leaf development during the reproductive transition. After SAM converts into IM, IM initiates three axillary meristems (AMs): branch meristems (BMs), spikelet meristems (SMs), or both, which further initiate floral meristems (FMs) (Koppolu and Schnurbusch, 2019). Specifically, BMs in the male inflorescence develop into long branches. Tassel with more branches contributes to prolonged pollination time and improved seed setting rate. However, more tassel branch numbers will consume more energy to produce pollen, thus reducing the absorption of nutrients by ear, and then affecting corn yield. Studies have found that smaller tassels with a few branches are beneficial to the increase of yield during modern maize breeding (Wang et al., 2020).

Maize, a monoecious crop, has a unique inflorescence structure consisting of primary and secondary paracladia (Vegetti and Anton, 1995). The development of inflorescence is a complex process, regulated by numerous genes. Several genes affecting tassel branch trait have been cloned in maize based on mutant analysis. For example, *FASCIATED EAR 4* (*FEA4*) affects the transition from SAM to IM, and its mutations cause more tassel branches (Pautler et al., 2015). The genes *ramosa1* (*ra1*), *ramosa2* (*ra2*), *ramosa3* (*ra3*) and *RAMOSA1 ENHANCER LOCUS2* (*rel2*), play important roles in regulating inflorescence branching and result in increased tassel branch number (TBN) (Vollbrecht et al., 2005; Satoh-Nagasawa et al., 2006; Gallavotti et al., 2010). In contrast, *unbranched2* (*ub2*), *unbranched3* (*ub3*) and *tasselsheath4* (*tsh4*), which belong to the SQUAMOSA PROMOTER BINDING PROTEIN-LIKE (SPL) family, regulate the differentiation of lateral primordia and have mutants that exhibit a significant reduction in TBN (Chuck et al., 2014). *KNOTTED1* (*KN1*) plays a crucial role in stem cell maintenance and organ initiation in shoot meristems, independent of the CLV-WUS pathway. *kn1* mutants in B73 background produce tassel primordia with fewer AMs, leading to fewer TBN (Kerstetter et al., 1997). BLH12 and BLH14, two cofactors of KN1, function redundantly in maintenance of axillary meristems and tassel branch patterning (Tsuda et al., 2017). Phytohormones also play significant roles in plant development and growth, including TBN. For example, *SPARSE INFLORESCENCE1* (*SPI1*) and *VANISHING TASSEL2* (*VT2*) participate in auxin biosynthesis, while *BARREN INFLORESCENCE1* (*BIF1*) and *BARREN INFLORESCENCE4* (*BIF4*) are involved in auxin signaling (Gallavotti et al., 2008; Phillips et al., 2011; Galli et al., 2015). Additionally, nutrition transport and epigenetic regulation are also verified to affect tassel morphology development (Chatterjee et al., 2014; Forestan et al., 2018).

The juvenile-to-adult phase transition is a critical biological process that signifies the transformation of SAM from the vegetative to the inflorescence stage. MicroRNA156 (miR156) is a class of conserved endogenous microRNAs that regulate gene expression post-transcriptionally in plants. It has been demonstrated that miR156 is involved in multiple essential biological processes, including the maintenance of the juvenile phase of plants and flower organ development (Wang and Wang, 2015). In Arabidopsis, prolonged juvenile vegetative periods and delayed flowering were observed as a result of constitutive miR156 expression (Wu and Poethig, 2006). Similarly, overexpression of *OsmiR156b* or *OsmiR156h* in rice caused a prolonged juvenile vegetative period and delayed flowering (Xie et al., 2006). In maize, the *Corngrass1* (*Cg1*) mutant exhibited a prolonged juvenile stage and retention of juvenile traits in the adult reproductive phase due to the overexpression of two tandem miR156 genes in the meristem and lateral organs (Chuck et al., 2007). miR156 is a class of microRNAs that are well-conserved and target a specific subset of SPLs in plants. These microRNAs define an age-dependent flowering regulatory pathway, known as the miR156-SPL modules, and have been verified to play important roles in multiple developmental processes in plants, such as the transition from the vegetative phase to the reproductive stage and the development of flower organs (Wang et al., 2009). In rice, mutation of *Ideal Plant Architecture1* (*IPA1*), which is targeted by miR156, led to altered plant architecture, including panicle branches (Jiao et al., 2010). Thus, miR156-SPL modules coordinately regulate plant growth and development traits in crops.

The maize mutant *Teopod2* (*Tp2*) has been extensively studied for over a century due to its dominant phenotype affecting tassel development. However, the genetic basis of *Tp2* and its interactions with other genes involved in tassel development are still unclear. In this study, we successfully cloned *Tp2* and mapped it to Chromosome 10, which encodes *zma-miR156h*, a paralog of *Cg1*. Overexpression of *zma-miR156h* resulted in the similarity of *Tp2* and *Cg1* phenotypes. Differential expression analysis and prediction of targeted genes identified several significantly downregulated SPL genes, including *ub2*, *ub3*, *tsh4* and *ZmSBP13/23/27/29*. The phenotypic investigation of CRISPR-edited *ZmSBP13* mutants provided conclusive evidence of its involvement in tassel development. The observed decrease in TBN clearly indicates that *ZmSBP13* is a target gene of *zma-miR156h*. Motif analysis of *ZmSBP13*-targeted DNA sequences indicated that it binds to typical regulatory sites involved in inflorescence architecture. These findings enhance our understanding of tassel branch development in maize and suggest that precise manipulation of miR156-SPL modules has significant potential to improve plant architecture and crop quality in modern breeding programs.

## 2 Materials and methods

### 2.1 Plant materials and trait measurements

The F_1_ population was generated by crossing the maize inbred line Mo17 as the female parent with *Tp2/Tp2* as the male parent during the winter season in Hainan, China. The F_1_ seeds were then grown in the winter of 2016 in Hainan, China and allowed to undergo self-fertilization to produce the F_2_ population. The agronomic traits, including plant height, ear height, length and width of ear leaf, and tassel branch number, were assessed for each individual after the flowering stage. A total of 177 individuals from two F_2_ families were subjected to chi-square test. One segregating F_2_ family consisting of 89 individuals was utilized for BSR-Seq (Bulked segregant RNA-Seq) (Liu et al., 2012) and MMAPPR (mutation mapping analysis pipeline for pooled RNA-seq) (Hill et al., 2013) analyses. Three OE lines, one CRISPR lines, and their respective negative controls were planted at the experimental station of Huazhong Agricultural University in Wuhan, China, and were managed according to local agronomic practices.

### 2.2 RNA isolation and gene expression analysis

To perform BSR-Seq and MMAPPR, total RNAs were extracted from two pooled samples of leaf tissues from 20 mutant individuals exhibiting single tassel branch number and 20 wild type individuals showing multiple tassel branch numbers, respectively. To detect differentially expressed genes, total RNAs were extracted from 2mm tassels of homozygous wild-type and homozygous mutants, respectively, and each set was prepared with two biological replicates. Additionally, miRNAs were extracted from the SAM of 10 homozygous wild-type and 10 homozygous mutants, respectively, to obtain the expression level of microRNA. The Direct-zol RNA Miniprep Kit (ZYMO RESEARCH) was used for total RNA and miRNA extraction according to the manufacturer’s instructions.

The raw PE (pair-end) sequencing data of mRNA-seq were first preprocessed using fastp (Chen et al., 2018) software to remove short reads (<72 bp) and low-quality base pair (q>20). over 81% clean reads were successfully mapped to the B73 RefGen_v4 using Tophat (version 2.1.1) (**Supplementary Table S1**). The expression level was calculated using Cufflinks (version 2.2.1) with FPKM (fragments per kilo bases of exon per million fragments mapped). Cuffdiff was then used to perform a differential expression analysis between wild type and *Tp2* mutant, using a base of |log2(Fold change)| ≥1 and false discovery rate (FDR) <0.001. To investigate the functions of the differential expression genes, GO enrichment analysis was performed using agriGO v2.0 (Tian et al., 2017).

To evaluate the expression level of small RNAs, the raw reads were first subjected to adaptor sequence and short read removal using Cutadapt (version 1.9.1). Over 92.6% of the clean reads (**Supplementary Table S2**) were retained and mapped to the Chr10.fa from the B73 RefGen_v4 reference genome. 80% of the clean data were randomly sampled 100 times, and the expression levels were calculated with RPM (reads of exon model per million mapped reads) using ShortStack (version 3.8.5). Expression differences were compared by extracting the RPM values of the zma-mir156h gene in the mutant and wild-type samples.

### 2.3 Quantitative real-time PCR

To validate the results of RNA-seq and miRNA-seq, we performed quantitative real-time PCR (qRT-PCR) to determine the expression levels of *Zm00001d025786* and *zma-miR156h*. For *Zm00001d025786*, cDNA synthesis was conducted using the HiScript® II 1st Strand cDNA Synthesis Kit, and RT-PCR was carried out using AceQ® Universal SYBR qPCR Master Mix according to the manufacturer’s instructions with three technical replicates. For *zma-miR156h*, cDNA synthesis was conducted using the miRNA 1st Strand cDNA Synthesis Kit (stem-loop method), and RT-PCR was carried out using the miRNA Universal SYBR qPCR Master Mix according to the manufacturer’s instructions with three technical replicates. The primers for *Zm00001d025786* and *zma-miR156h* were designed and listed in **Supplementary Table S3**. We used ubiquitin as an internal control for normalization, and relative expression levels were evaluated using the 2^-ΔΔCT method.

### 2.4 Vector construction and plant transformations

To construct the overexpression vector, the pre-MIR156h sequence was amplified using specific primers listed in **Supplementary Table S4**. The resulting PCR product was then cloned into the vector pCombia3300-3xflag, and subsequently transformed into the maize inbred line B73 by Beijing BomeiXingao Technology Co., Ltd. The CR-*sbp13* vector construction and plant transformation were carried out by Wimi Biotechnology Co., Ltd (Changzhou, China).

### 2.5 tsCUT&Tag experiment

A tsCUT&Tag experiment was conducted for the identification of regulatory network following the guideline of our previous study (Wu et al., 2022). The raw reads from two replicates were subjected to trimming using Fastqc (version 0.11.5). The clean reads were then mapped to the B73 RefGen_v4 genome using Bowtie2 (version 2.4.1) with the parameters “–no-mixed –no-discordant”. Duplicated reads were removed using SAMtools (version 1.9), and peak calling was performed using MACS2 (version 2.2.7.1) with parameters “-g 2.2e+9 -p 1e-5”. Finally, peak genes were obtained using the BEDTools (version 2.27) windows command. Additionally, motif analysis was performed using MEME-ChIP (Machanick and Bailey, 2011).

## 3 Results

### 3.1 *Tp2* affects multiple traits of maize development

**Figure 1:**
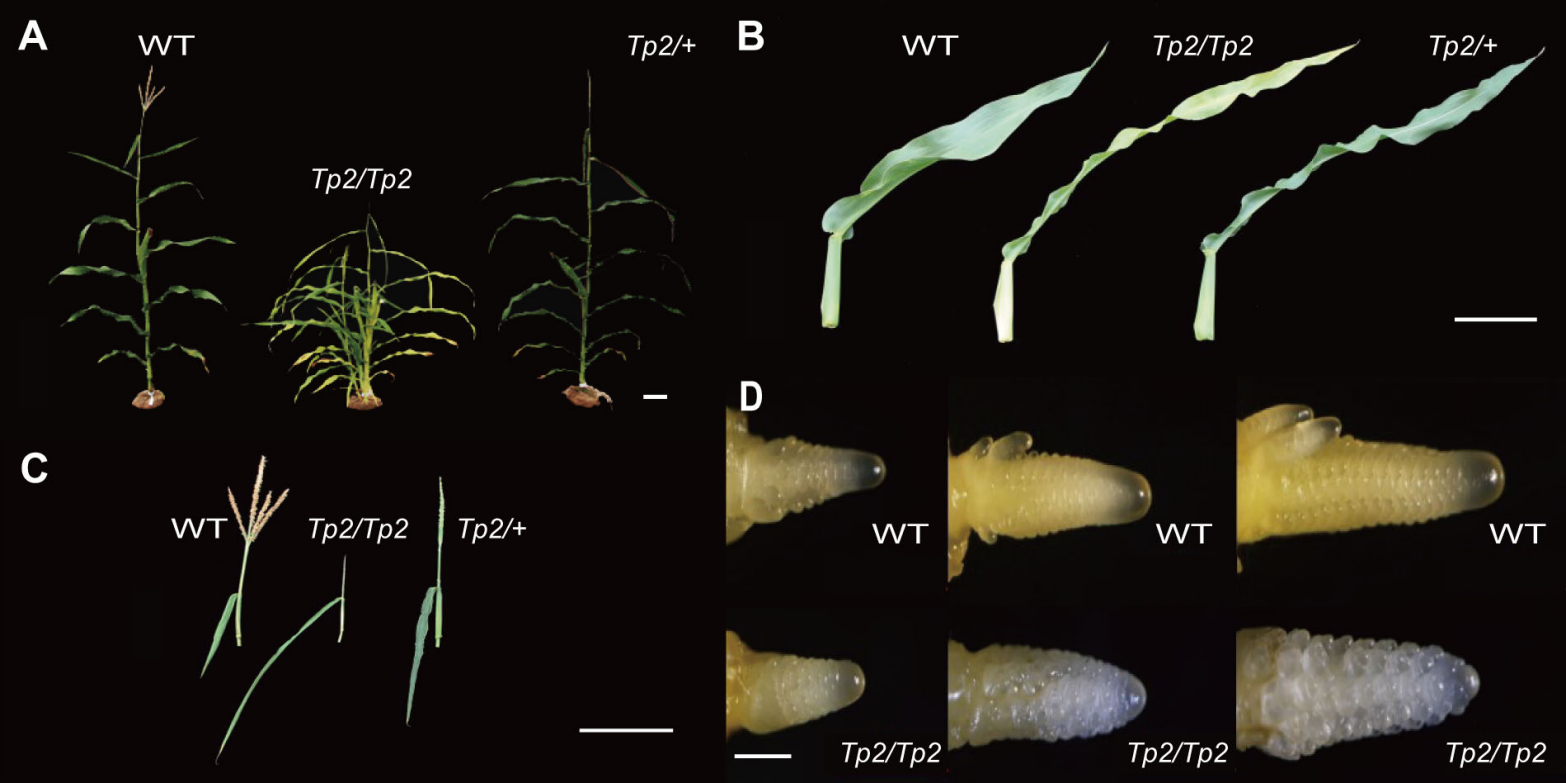
Vegetative and inflorescence traits. In each, the wild type is on the left, *Tp2/Tp2* is on the middle and *Tp2/+* is on the right. **(A)** Whole plant during vegetative development. **(B)** Excised mature leaf blades. **(C)** Mature tassels. **(D)** 1-3mm tassel of wild type and *Tp2/Tp2*. Scale bars= 10 cm in **(A–C)** 0.5 mm in **(D)**.

*Tp2* is a classical mutant from Maize Genetics Cooperation Stock Center that extends the duration of the juvenile vegetative phase of maize development. In normal maize plants, tillers that grow in axillary positions of juvenile leaves are produced in low numbers, ranging from zero to a few (Chuck et al., 2007). However, *Tp2* mutants exhibit an increased number of phytomers that produce ears and initiate numerous tillers in the axils of each leaf, resulting in a larger number of leaves that retain a slender juvenile morphology (middle) (**Figures 1A, B**). While *Tp2/+* plants initiate tassels later than their wild-type siblings, the mutant tassel arrests differentiation at the same time as, or shortly before, the primary meristem of a wild-type tassel completes its development, resulting in the formation of a single tassel branch (**Figure 1C**). To assess the impact of the mutation on other agronomic traits, such as plant height (PH), ear height (EH), ear leaf length (ELL), and ear leaf width (ELW), we measured these traits in homozygous wild-type and homozygous mutant plants. An independent sample Student’s *t*-test was performed to evaluate the significance of the difference between the two counterpart genotypes. The phenotypic statistics revealed significant differences for most agronomically important traits, including plant height, ear height, ear leaf length, and ear leaf width, when compared to the wild type (**Supplementary Figures S1A, B**). Furthermore, the TBN phenotypes of *Tp2/Tp2* and *Tp2/+* and their response to variation in gene dose indicate that *Tp2* is characterized by gain-of-function mutations. To better understand the period during which the mutant tassels exhibit abnormal differentiation, we examined the growth process of young ears ranging from 1-3mm in size. Our observations suggest that the differentiation of mutant tassels begins to deviate from normalcy around 2mm, as revealed by microscopic analysis (**Figure 1D**).

### 3.2 Genetic analysis and mapping of the *Tp2* mutant

To explore the genetic basis of TBN variation in the *Tp2* mutant, the maize inbred Mo17 was used as the female parent to cross with *Tp2* mutant as the male parent, which generated two segregating F_2_ families comprising 177 plants. The segregation ratio of single tassel branch to multiple tassel branches in the two F_2_ family were consistent with a 3:1 ratio (**Supplementary Table S5**), thereby supporting the hypothesis that a single, dominant gene is responsible for the TBN phenotype.

**Figure 2:**
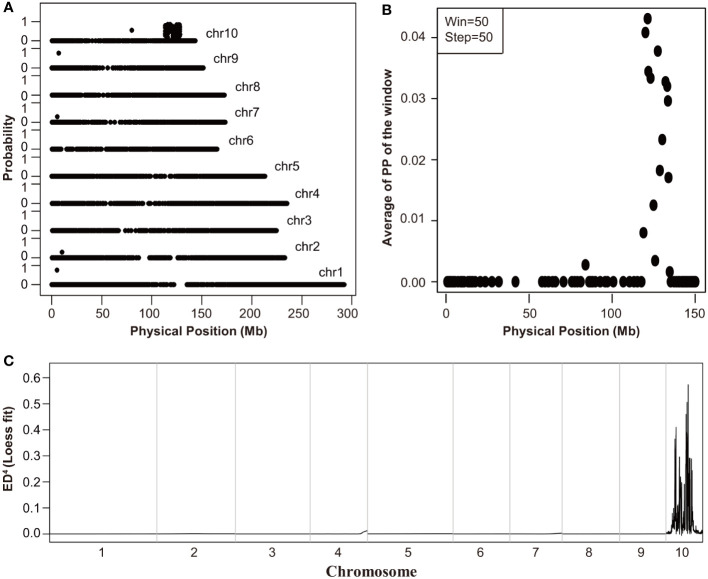
The initial mapping of *Tp2*. **(A, B)** The mapping results of BSR-Seq. The physical position of each SNP marker was plotted versus the probability of each SNP marker being in complete linkage disequilibrium with the causal gene **(A)** and the Chromosome 10 was scanned by using a window containing 50 SNPs with a step size of 50 SNPs **(B)**. **(C)** The mapping results of MMAPPR. Loess fit curve calculated using the ED^4^ (Euclidean distance raised to the fourth power) scores across the genome.

**Figure 3:**
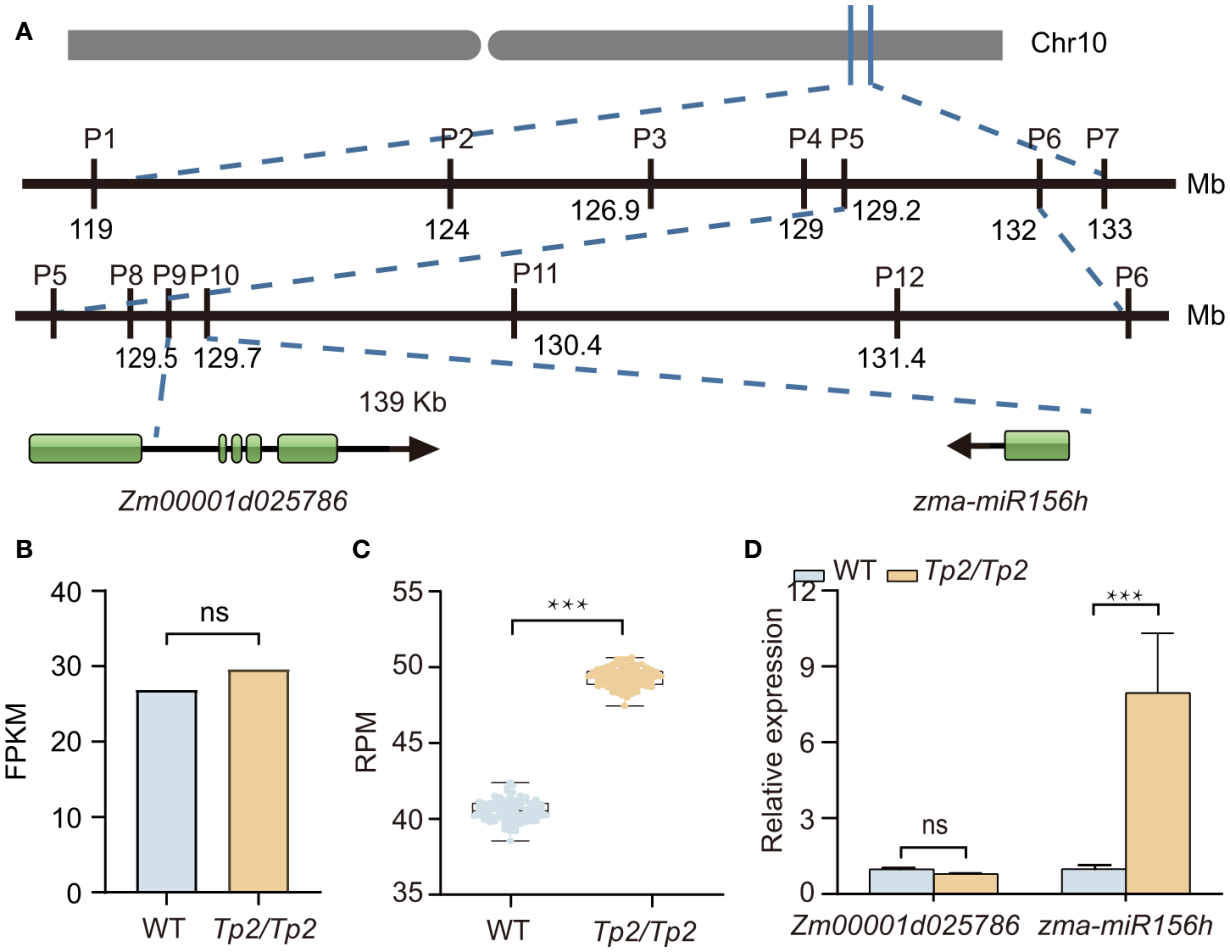
Fine mapping and candidate genes analysis for *Tp2*. **(A)** Fine mapping of *Tp2* on maize chromosome 10. The physical positions of molecular markers (according to the B73_V4) used for fine mapping of the *Tp2* locus. **(B)** Transcription levels of *Zm00001d025786* estimated by mRNA-seq in WT (wild type) and the *Tp2/Tp2* mutant from the about 2mm tassel tissue. **(C)** Transcription levels of *zma-miR156h* estimated by miRNA-Seq in WT and the *Tp2/Tp2* mutant from the SAM tissues. **(D)** Relative expression levels of *Zm00001d025786* and *zma-miR156h* in homozygous wild-type and homozygous mutant via qRT-PCR assay, respectively. P value was determined by Student’s t-test. ***P < 0.001, “ns” means it is no significant difference.

To identify the causal gene of *Tp2* mutant, we bulked RNA samples from mutant and non-mutant individuals of a F_2_ population into two separate pools (wild-type pool and mutant pool) and performed RNA-Seq. We used BSR-Seq and MMAPPR to delimit the candidate interval of the causal gene. BSR-Seq and MMAPPR mapping analysis pipeline identified a genomic interval of approximately 14 Mb, between 119 and 133 Mb on chromosome 10 as the location of the *Tp2* mutant (**Figures 2A–C**). We selected seven SNP markers (**Supplementary Table S6**) within the candidate interval (119 Mb to 133 Mb) to genotype a large segregating population, including approximately 5,000 individuals. Based on the genotypes and phenotypes of the progeny families derived from recombinants, Tp2 was localized between the markers P5 (129.2 Mb) and P6 (132 Mb) (**Figure 3A**). To further fine-map *Tp2*, we genotyped the segregating population and the progeny families derived from recombinants using five newly developed markers (**Supplementary Table S6**). Based on the genotypic and phenotypic data, we narrowed down the location of *Tp2* to an approximately 139-kb region flanked by markers P9 and P10, in which only two genes (*Zm00001d025786* and *zma-miR156h*) are located (**Figure 3A**).

To mine the candidate genes for *Tp2*, we calculated gene abundance from transcriptome data. Since the tassel of *Tp2/Tp2* begin to appear abnormal at about 2mm, SAM is the key niche for the growth and development of the aerial part. Therefore, the RNA library from 2mm tassel and the SAM of 2-week-old seedling of homozygous wild-type and homozygous mutant genotypes were used for mRNA sequencing and miRNA sequencing, respectively. For the data of mRNA sequencing, the Spearman Correlation coefficients are at least 81% between two biological replicates (**Supplementary Figures S2A, B**), and the DEG analysis revealed a total of 58 genes were up-regulated and 43 genes were down-regulated (**Supplementary Figure S2C; Supplementary Table S7**). To further explore the functions of the DEGs, we performed gene ontology (GO) enrichment analysis (*p*-value < 0.01). The significantly enriched biological process such as plant organ development and formation, regulation of organ morphogenesis, flower development (**Supplementary Figure S3**). These findings show that these DEGs are related with regulating maize inflorescences and organ morphogenesis.

Notably, the expression level of *Zm00001d025786* had no difference between wild type and mutant pools (**Figure 3B**). Further miRNA sequencing between mutant and wild-type showed that mainly 20–24 nucleotide noncoding RNAs were enriched (**Supplementary Figure S4**), indicating that the high quality of sequencing data. Further analysis of miRNA sequencing data showed the expression level of *zma-miR156h* in *Tp2/Tp2* is up-regulated (**Figure 3C**). To validate the results of the transcriptome sequencing, quantitative Real-Time Polymerase Chain Reaction (qRT-PCR) was performed. The expression levels of *Zm00001d025786* and *zma-miR156h* genes were consistent with the results of transcriptome sequencing (**Figure 3D**). Thus, *zma-miR156h* was considered as the candidate gene of *Tp2* mutant

### 3.3 *zma-miR156h*-OE and CR-*sbp13* plants produce similar phenotypes

**Figure 4:**
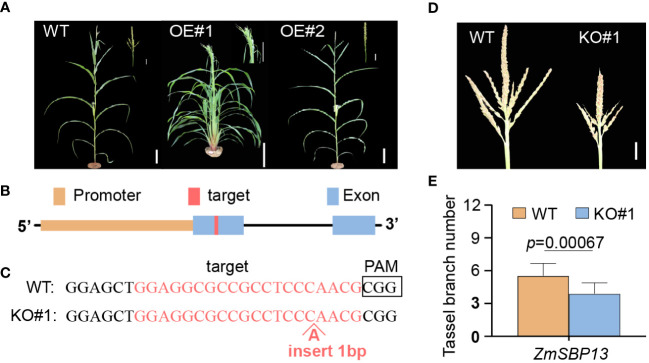
Characters of transgenic *zma-miR156h*-OE and CR-*sbp13* plants. **(A)** Plant morphology from wild-type (left) and overexpression transgenic (OE#1 and OE#2) plants after the flowering stage. **(B)** Gene structure of *ZmSBP13* and the target site in the first exon of *ZmSBP13* for CRISPR/Cas9 editing. **(C)** Sequences of a homozygous knockout line with insertion in target site (KO#1). The wild-type sequence is shown at the Top. Target site and protospacer-adjacent motif (PAM) sequences are highlighted and boldface fonts, respectively. **(D)** Morphologies of wild-type and knockout line. **(E)** Phenotypic comparison of wild-type and knockout line. The data is shown as means ± SD; Scale bars= 10 cm in **(A)** 20 mm in **(D)** *P* value was determined by Student’s t test.

MicroRNA156 plays vital roles in maize development and reproduction. Vegetative phase change in maize is regulated by miR156, a microRNA that promotes the expression of the juvenile phase and represses the expression of the adult phase. To confirm that overexpression of *zma-miR156h* is the cause of the *Tp2* phenotype, we generated transgenic maize lines using the B73 as the background, in which a 115-bp precursor sequence of *zma-miR156h* was overexpressed by using the ubiquitin promoter (**Supplementary Figure S5**). We observed conspicuous single tassel branch in *zma-miR156h*-OE compared with wild-type. Additionally, in place of tassel branches, long bract leaves were present, clustered at the base of the tassel in *zma-miR156h*-OE#1 (**Figure 4A**). The relative expression level of *miR156* was compared between the OE-plants and their control. Compared with the wild type counterpart, *miR156* were up-regulated in the OE lines. The expression levels and phenotypic results of OE#1 and OE#2 revealed that the pleiotropic phenotype in OE-plants was positively correlated with the expression level of *miR156* (**Supplementary Figure S6**), which was related to the regulation of multiple pathways by *miR156*. The overexpression of *zma-miR156h* in maize plants produced similar phenotypes to *Tp2* mutants, indicating that the *Tp2* mutant phenotype is due to overexpression of *zma-miR156h*.

Studies have shown that miR156 regulates the spatiotemporal changes of plants by repressing SPL genes (Wang et al., 2009; Wu et al., 2009). To further elucidate the molecular bases of the defects in plant architecture caused by overexpression of *zma-miR156h*, we analyzed the target genes of *zma-miR156h* based on the maize cDNA library (*zea mays*, cDNA, AGPv4) using the web-based plant small RNA target analysis tool psRNATarget (Dai and Zhao, 2011). A total of 61 potential miR156-targeting genes were obtained (**Supplementary Table S8**). Furthermore, the functional annotation of 43 down-regulated genes in *Tp2* mutant showed that 7 genes were annotated to encode SBP-domain protein, and all of them were target genes of miR156 (**Supplementary Table S9**). Among them, *tsh4*, *ub2* and *ub3* had already been reported to affect the development of tassel branch in previous studies, while the other four SPL genes (*ZmSBP13*, *ZmSBP23*, *ZmSBP27*, *ZmSBP29*) have not been researched yet.

To further investigate the relationship between SPL genes and *zma-miR156h*, we chose *ZmSBP13* gene to generate knockout transgenic line (CR-*sbp13*) using CRISPR-Cas9 (**Figures 4B, C**). Using a PCR-based genotyping procedure, we identified an independent *sbp13* knockout line with a 1bp insertion in the *ZmSBP13* gene body in the T1 generation. Phenotypic and statistical analyses showed that TBN was decreased in these CR-*sbp13* lines compared with WT plants (**Figures 4D, E**). The CR-*sbp13* transgenic lines produced similar phenotypes with *zma-miR156h*-OE, indicating that *ZmSBP13* gene, as the target of *zma-mir156h*, was inhibited by *zma-mir156h*, which led to the decrease of the tassel branches number.

### 3.4 *ZmSBP13* may target multiple genes to regulate inflorescence structure

**Figure 5:**
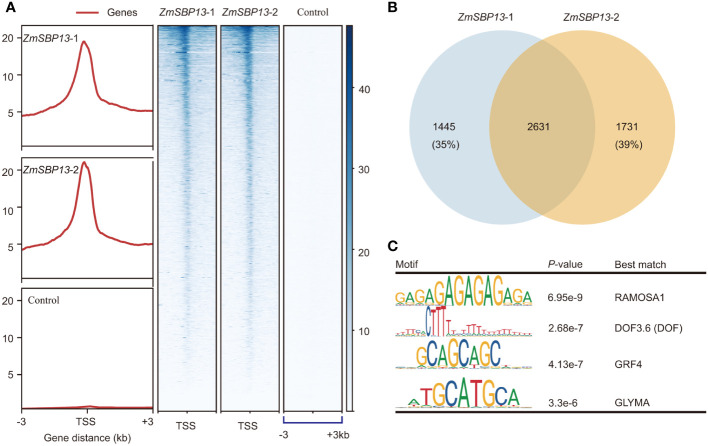
*ZmSBP13*-bound target genes. **(A)** Heatmap of the binding sites of *ZmSBP13* at the positions − 3.0 kb upstream to + 3.0 kb downstream relative to transcription start site (TSS). **(B)** Overlapped target genes between *ZmSBP13* tsCUT&Tag replicates. **(C)** *ZmSBP13* bound motifs identified by MEME. The motif sequence is shown in the left column, the *P* value is shown in middle column and the corresponding proteins are shown in the right column.

To investigate the molecular mechanism in regulating TBN, we clarified the potential target genes of *ZmSBP13* using tsCUT&Tag assay. The Spearman Correlation coefficients for tsCUT&Tag data of two replicates is 0.98 (**Supplementary Figure S7A**). And *ZmSBP13*-bound signals were more prominent at promoter regions compared with negative control (**Figure 5A**; **Supplementary Figures S7B, C**). The number of overlapping detectable target genes for *ZmSBP13* were 2631 (**Figure 5B**; **Supplementary Table S10**), demonstrating a good reproducibility of the data from tsCUT&Tag. To reveal the proteins that potentially interact with *ZmSBP13*, we performed motif analysis of *ZmSBP13*-targeted DNA sequences obtained from tsCUT&Tag results. Notably, the motif analysis showed that it is the typical binding site for the RAMOSA1, DOF, GROWTH REGULATING FACTOR (GRF4) and GLYMA proteins which control inflorescence architecture (**Figure 5C**), indicating that *ZmSBP13* may interact with multiple proteins to regulate inflorescence structure.

## 4 Discussion

*Tp2* had been verified that exhibits different phenotypes under different genetic backgrounds, indicating that gene dosage contributes to the observed variation (Poethig, 1988). This is further supported by our findings that overexpression of *zma-miR156h* produced different phenotypes (**Figure 4A**). Another dominant mutant, *Cg1*, exhibited more severe phenotypes compared to several Teopod mutants (Chuck et al., 2007). *Cg1* encodes two tandem miR156 genes (*miR156b/c*) that are overexpressed in the meristem and lateral organs, while overexpression of *zma-miR156h* produced similar phenotypes to *Cg1*. Both mutants result from overproduction and misexpression of mature miR156, highlighting the importance of suitable expression of miRNA156 throughout the plant’s life cycle. *Teopod1* and *Teopod3* also exhibited similar phenotypes to *Tp2*, including an increase in the number of vegetative phytomers and phytomers producing ears, tillers, and prop roots, a decrease in the size of leaves, internodes, ear, and tassel, and transformation of reproductive structures into vegetative ones (Poethig, 1988). Additionally, *glossy15* (*gl15*) also affected the juvenile-to-adult phase transition but appeared to affect only epidermal phase transition traits, such as epidermal waxes, and acted downstream of *Cg1*, *Tp1*, and *Tp2* (Moose and Sisco, 1994). *Gl15* encodes a member of the AP2-domain family and functions in maintaining juvenile epidermal traits by negatively regulating microRNA172, which has been shown to promote *juvenile-to-adult* phase transition (Moose and Sisco, 1996; Lauter et al., 2005).

RNA-seq analysis revealed several differentially expressed genes associated with maize inflorescence development in *Tp2* mutants. Among them, genes such as *ub2*, *ub3*, *tsh4*, *ra3*, and *silky1* have been previously demonstrated to affect maize inflorescence development (Ambrose et al., 2000; Satoh-Nagasawa et al., 2006; Chuck et al., 2014). Additionally, MADS-box and SPL family genes, including *ZmMADS8/14/18/31* and *ZmSBP13/23/27/29* were significantly differentially expressed between *Tp2* mutants and wild type materials (**Supplementary Table S7**). These genes have been reported to play important roles in inflorescence development in maize and other species (Heuer et al., 2000; Heuer et al., 2001; Schilling et al., 2018). *Thiamine biosynthesis1* (*Thi1*) also exhibited significant up-regulated expression in *Tp2* mutants, whose paralogue *thiamine biosynthesis2* has been shown to affect maintenance of SAM and inflorescence development in maize (**Supplementary Table S7**) (Woodward et al., 2010). Furthermore, two gibberellin synthesis genes, *dwarf plant3* and *gibberellin 20-oxidase1*, were significantly down-regulated in *Tp2* mutants, possibly explaining relatively decreased plant height phenotype of *Tp2* (**Supplementary Table S7**) (Winkler and Helentjaris, 1995; Voorend et al., 2016). Thus, dissection of *Tp2* mutants provides insights into the understanding of inflorescence and plant architecture development in maize.

Maize tassel branch trait is regulated by multiple genes and involved in various regulatory networks. The CLV-WUS feedback pathway plays an essential role in maintaining and determining the SAM. *THICK TASSEL DWARF1* (*TD1*), *FASCIATED EAR2* (*FEA2*) and *ZmCLE7* are some of the primary genes involved in this pathway (Taguchi-Shiobara et al., 2001; Bommert et al., 2005; Liu et al., 2021). The ramosa pathway, centered around the REL2/RA1 transcriptional repressor complex, plays an antagonizes role in the formation of indeterminate branches during maize inflorescence development. This pathway includes *ra2* and *ra3* (Gallavotti et al., 2010). The miR156-SPL pathway also plays a crucial role in tassel branch development by regulating Inflorescence bracts and axillary meristem growth, including *ub2*, *ub3*, *tsh4*, and *ZmSBP13* (identified in this study). Phytohormones, such as auxin, also play a vital role in regulating tassel developments, with *SPI1*, *VT2*, *BIF1* and *BIF4* genes having been identified in this regard (Gallavotti et al., 2008; Phillips et al., 2011; Galli et al., 2015). Epigenetic regulation also has been shown to affect tassel morphology development with histone deacetylase 108 (HDA108) playing a role in overall plant architecture and inflorescence patterning and fertility (Forestan et al., 2018). Crosstalks between different pathways can help to fully understand the mechanism of tassel development. Several genes, including *ra3*, *dwarf plant3*, *GA20ox1* and *thi1* exhibited significantly differentially expressed in *Tp2* mutants, providing an avenue for understanding the crosstalks between the miR156-SPL pathway and other pathways in tassel development.

During the process of modern maize breeding, the plant architecture of maize was significantly changed for adaptation to high planting density, including more compact architecture with reduced relative ear height and upper leaf angle, smaller tassel with decreased tassel branch number. In this study, we identified new miR156-SPL modules functioning in plant architecture development in maize. Overexpression of *zma-miR156h*, as well as knockout of *ZmSBP13*, exhibited decreased tassel branch number in various degrees. Therefore, the accurate regulation of miR156-SPL modules exhibited huge potential for plant architecture improvement during modern maize breeding.

Other than maize, the miR156-SPL modules are widely conserved in various plant species and plays crucial roles in multiple developmental processes throughout the entire life cycle. Overexpression of miR156 in plants, such as Arabidopsis, rice, tomato, among others, has been shown to cause prolonged juvenile vegetative periods and delayed flowering (Wu and Poethig, 2006; Xie et al., 2006). In rice, *Ideal Plant Architecture1* (*IPA1*) encodes the SPL family transcription factor, *OsSPL14*, which has been identified as a key regulator of plant architecture. Point mutations in the miRNA156 recognition site led to decreased tiller number and increased plant height and panicle branches due to the repression of *IPA1* mRNA degradation (Jiao et al., 2010; Miura et al., 2010; Lu et al., 2013). Additionally, miR156-SPL modules also functioned in grain filling (Wang et al., 2012), branch angle controlling (Ishii et al., 2013; Zhu et al., 2013), fruit ripening (Manning et al., 2006), biotic and abiotic stress responses (Stone et al., 2005; Padmanabhan et al., 2013; Stief et al., 2014). Subtle manipulation of miR156-SPL modules has partially contributed to plant architecture improvement, yield and quality elevation in rice, and will continually make great contributions during modern crop breeding processes including maize, wheat and so on.

**Figure 6:**
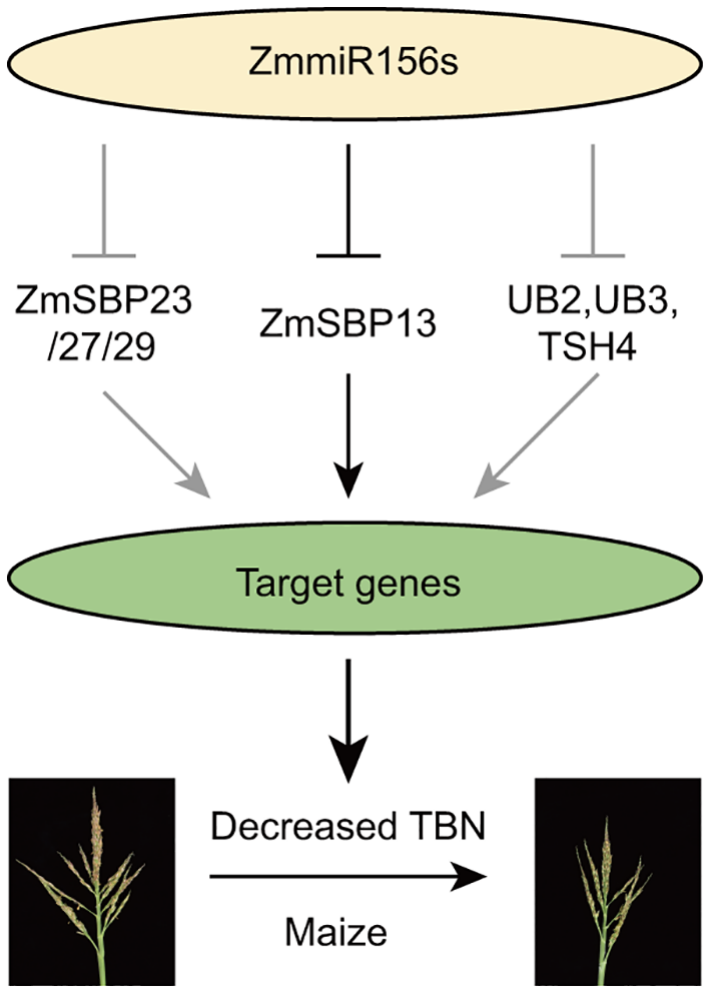
A proposed model for miRNA156-SPL to regulate the tassel branch.

Based on our results and previous studies, we proposed a miR156-SPL model regulating tassel branch development in maize (**Figure 6**): Overexpression of *zma-miR156h* in *Tp2* mutant, repressed the expression of series of SPL transcriptional factors, including *ZmSBP13*, *ZmSBP23/27/29*, as well as *UB2*, *UB3* and *TSH4*, which positively regulating a large number of target genes, therefore, contributing to decreased tassel branch number trait in maize. Especially, a new identified SPL gene, *ZmSBP13* affected tassel development by targeting many important genes in maize, for example *indeterminate spikelet1* (*ids1*), *KNOTTED1* (*KN1*), *ZmBLH12* and so on (**Supplementary Table S10**).

In summary, we characterized and functionally analyzed a classical maize dominant mutant, *Teopod2* (*Tp2*), and identified a microRNA, *zma-miR156h*, which regulates multiple agronomic traits in maize by inhibiting the expression of *ZmSBP13*, and the *ZmSBP13* may interact with multiple proteins to regulate inflorescence structure in maize. Nevertheless, there are still some unresolved questions, such as the relationships between *ZmSBP13* and other known SPL family genes (*ub2*, *ub3* and *tsh4*). *ub2*, *ub3* and *tsh4* had been verified functioning redundantly in tassel development (Chuck et al., 2014). Whether there are functional redundancies between *ZmSBP13* and three known SPL genes, as well as other unknown SPL genes, should be addressed and dissected by future genetic analyses. Moreover, overexpression of miRNA156 in various species exhibiting multiple phenotypes, including plant architecture, inflorescence structure, and yield. Realizing accurate regulation of the miRNA156-SPL module remains a significant challenge, as well as an essential measure to satisfy the increasing demands of cereals.

## Data availability statement

The datasets presented in this study can be found in National Center for Biotechnology Information. The names of the accession number(s) can be found below: SRR23581318, SRR23581319, SRR23581320, SRR23581321, SRR23581322.

## Author contributions

LL and JL designed the experiments. JL conducted experiments with the assistance of XW. JL generated the datasets and performed data analysis with the assistance of JW. JL and XW wrote the manuscript; LL and JL reviewed and edited the manuscript. All authors have read and agreed to the published version of the manuscript.

## References

[B1] Ambrose B. A. Lerner D. R. Ciceri P. Padilla C. M. Yanofsky M. F. Schmidt R. J. (2000). Molecular and genetic analyses of the silky1 gene reveal conservation in floral organ specification between eudicots and monocots. Mol. Cell 5, 569–579. doi: 10.1016/s1097-2765(00)80450-5 10882141

[B2] Bommert P. Lunde C. Nardmann J. Vollbrecht E. Running M. Jackson D.. (2005). Thick tassel dwarf1 encodes a putative maize ortholog of the arabidopsis CLAVATA1 leucine-rich repeat receptor-like kinase. Development 132, 1235–1245. doi: 10.1242/dev.01671 15716347

[B3] Chatterjee M. Tabi Z. Galli M. Malcomber S. Buck A. Muszynski M.. (2014). The boron efflux transporter ROTTEN EAR is required for maize inflorescence development and fertility. Plant Cell 26, 2962–2977. doi: 10.1105/tpc.114.125963 25035400 PMC4145125

[B4] Chen S. Zhou Y. Chen Y. Gu J. (2018). Fastp: an ultra-fast all-in-one FASTQ preprocessor. Bioinformatics 34, i884–i890. doi: 10.1093/bioinformatics/bty560 30423086 PMC6129281

[B5] Chuck G. S. Brown P. J. Meeley R. Hake S. (2014). Maize SBP-box transcription factors unbranched2 and unbranched3 affect yield traits by regulating the rate of lateral primordia initiation. Proc. Natl. Acad. Sci. U.S.A. 111, 18775–18780. doi: 10.1073/pnas.1407401112 25512525 PMC4284592

[B6] Chuck G. Cigan A. M. Saeteurn K. Hake S. (2007). The heterochronic maize mutant Corngrass1 results from overexpression of a tandem microRNA. Nat. Genet. 39, 544–549. doi: 10.1038/ng2001 17369828

[B7] Dai X. Zhao P. X. (2011). psRNATarget: a plant small RNA target analysis server. Nucleic Acids Res. 39, W155–W159. doi: 10.1093/nar/gkr319 21622958 PMC3125753

[B8] Forestan C. Farinati S. Rouster J. Lassagne H. Lauria M. Dal Ferro N.. (2018). Control of maize vegetative and reproductive development, fertility, and rRNAs silencing by HISTONE DEACETYLASE 108. Genetics 208, 1443–1466. doi: 10.1534/genetics.117.300625 29382649 PMC5887141

[B9] Gallavotti A. Barazesh S. Malcomber S. Hall D. Jackson D. Schmidt R. J.. (2008). Sparse inflorescence1 encodes a monocot-specific YUCCA-like gene required for vegetative and reproductive development in maize. Proc. Natl. Acad. Sci. U.S.A. 105, 15196–15201. doi: 10.1073/pnas.0805596105 18799737 PMC2567514

[B10] Gallavotti A. Long J. A. Stanfield S. Yang X. Jackson D. Vollbrecht E.. (2010). The control of axillary meristem fate in the maize ramosa pathway. Development 137, 2849–2856. doi: 10.1242/dev.051748 20699296 PMC2938917

[B11] Galli M. Liu Q. Moss B. L. Malcomber S. Li W. Gaines C.. (2015). Auxin signaling modules regulate maize inflorescence architecture. Proc. Natl. Acad. Sci. U.S.A. 112, 13372–13377. doi: 10.1073/pnas.1516473112 26464512 PMC4629326

[B12] Godfray H. C. J. Beddington J. R. Crute I. R. Haddad L. Lawrence D. Muir J. F.. (2010). Food security: the challenge of feeding 9 billion people. Science 327, 812–818. doi: 10.1126/science.1185383 20110467

[B13] Heuer S. Hansen S. Bantin J. Brettschneider R. Kranz E. Lörz H.. (2001). The maize MADS box gene ZmMADS3 affects node number and spikelet development and is co-expressed with ZmMADS1 during flower development, in egg cells, and early embryogenesis. Plant Physiol. 127, 33–45. doi: 10.1104/pp.127.1.33 11553732 PMC117960

[B14] Heuer S. Lörz H. Dresselhaus T. (2000). The MADS box gene ZmMADS2 is specifically expressed in maize pollen and during maize pollen tube growth. Sex Plant Reprod. 13, 21–27. doi: 10.1007/PL00009838

[B15] Hill J. T. Demarest B. L. Bisgrove B. W. Gorsi B. Su Y.-C. Yost H. J. (2013). MMAPPR: mutation mapping analysis pipeline for pooled RNA-seq. Genome Res. 23, 687–697. doi: 10.1101/gr.146936.112 23299975 PMC3613585

[B16] Ishii T. Numaguchi K. Miura K. Yoshida K. Thanh P. T. Htun T. M.. (2013). OsLG1 regulates a closed panicle trait in domesticated rice. Nat. Genet. 45, 462–465. doi: 10.1038/ng.2567 23435087

[B17] Jiao Y. Wang Y. Xue D. Wang J. Yan M. Liu G.. (2010). Regulation of OsSPL14 by OsmiR156 defines ideal plant architecture in rice. Nat. Genet. 42, 541–544. doi: 10.1038/ng.591 20495565

[B18] Kerstetter R. A. Laudencia-Chingcuanco D. Smith L. G. Hake S. (1997). Loss-of-function mutations in the maize homeobox gene, knotted1, are defective in shoot meristem maintenance. Development 124, 3045–3054. doi: 10.1242/dev.124.16.3045 9272946

[B19] Koppolu R. Schnurbusch T. (2019). Developmental pathways for shaping spike inflorescence architecture in barley and wheat. J. Integr. Plant Biol. 61, 278–295. doi: 10.1111/jipb.12771 30609316

[B20] Lauter N. Kampani A. Carlson S. Goebel M. Moose S. P. (2005). microRNA172 down-regulates glossy15 to promote vegetative phase change in maize. Proc. Natl. Acad. Sci. U.S.A. 102, 9412–9417. doi: 10.1073/pnas.0503927102 15958531 PMC1166634

[B21] Liu L. Gallagher J. Arevalo E. D. Chen R. Skopelitis T. Wu Q.. (2021). Enhancing grain-yield-related traits by CRISPR–Cas9 promoter editing of maize CLE genes. Nat. Plants 7, 287–294. doi: 10.1038/s41477-021-00858-5 33619356

[B22] Liu S. Yeh C.-T. Tang H. M. Nettleton D. Schnable P. S. (2012). Gene mapping via bulked segregant RNA-seq (BSR-seq). PloS One 7, e36406. doi: 10.1371/journal.pone.0036406 22586469 PMC3346754

[B23] Lu Z. Yu H. Xiong G. Wang J. Jiao Y. Liu G.. (2013). Genome-wide binding analysis of the transcription activator IDEAL PLANT ARCHITECTURE1 reveals a complex network regulating rice plant architecture. Plant Cell 25, 3743–3759. doi: 10.1105/tpc.113.113639 24170127 PMC3877814

[B24] Machanick P. Bailey T. L. (2011). MEME-ChIP: motif analysis of large DNA datasets. Bioinformatics 27, 1696–1697. doi: 10.1093/bioinformatics/btr189 21486936 PMC3106185

[B25] Manning K. Tör M. Poole M. Hong Y. Thompson A. J. King G. J.. (2006). A naturally occurring epigenetic mutation in a gene encoding an SBP-box transcription factor inhibits tomato fruit ripening. Nat. Genet. 38, 948–952. doi: 10.1038/ng1841 16832354

[B26] Miura K. Ikeda M. Matsubara A. Song X.-J. Ito M. Asano K.. (2010). OsSPL14 promotes panicle branching and higher grain productivity in rice. Nat. Genet. 42, 545–549. doi: 10.1038/ng.592 20495564

[B27] Moose S. P. Sisco P. H. (1994). Glossy15 controls the epidermal juvenile-to-Adult phase transition in maize. Plant Cell 6, 1343–1355. doi: 10.1105/tpc.6.10.1343 12244224 PMC160525

[B28] Moose S. P. Sisco P. H. (1996). Glossy15, an APETALA2-like gene from maize that regulates leaf epidermal cell identity. Genes Dev. 10, 3018–3027. doi: 10.1101/gad.10.23.3018 8957002

[B29] Padmanabhan M. S. Ma S. Burch-Smith T. M. Czymmek K. Huijser P. Dinesh-Kumar S. P. (2013). Novel positive regulatory role for the SPL6 transcription factor in the n TIR-NB-LRR receptor-mediated plant innate immunity. PloS Pathog. 9, e1003235. doi: 10.1371/journal.ppat.1003235 23516366 PMC3597514

[B30] Pautler M. Eveland A. L. LaRue T. Yang F. Weeks R. Lunde C.. (2015). FASCIATED EAR4 encodes a bZIP transcription factor that regulates shoot meristem size in maize. Plant Cell 27, 104–120. doi: 10.1105/tpc.114.132506 25616871 PMC4330574

[B31] Phalan B. Onial M. Balmford A. Green R. E. (2011). Reconciling food production and biodiversity conservation: land sharing and land sparing compared. Science 333, 1289–1291. doi: 10.1126/science.1208742 21885781

[B32] Phillips K. A. Skirpan A. L. Liu X. Christensen A. Slewinski T. L. Hudson C.. (2011). Vanishing tassel2 encodes a grass-specific tryptophan aminotransferase required for vegetative and reproductive development in maize. Plant Cell 23, 550–566. doi: 10.1105/tpc.110.075267 21335375 PMC3077783

[B33] Poethig R. S. (1988). Heterochronic mutations affecting shoot development in maize. Genetics 119, 959–973. doi: 10.1093/genetics/119.4.959 17246439 PMC1203479

[B34] Satoh-Nagasawa N. Nagasawa N. Malcomber S. Sakai H. Jackson D. (2006). A trehalose metabolic enzyme controls inflorescence architecture in maize. Nature 441, 227–230. doi: 10.1038/nature04725 16688177

[B35] Schilling S. Pan S. Kennedy A. Melzer R. (2018). MADS-box genes and crop domestication: the jack of all traits. J. Exp. Bot. 69, 1447–1469. doi: 10.1093/jxb/erx479 29474735

[B36] Stief A. Altmann S. Hoffmann K. Pant B. D. Scheible W.-R. Bäurle I. (2014). Arabidopsis miR156 regulates tolerance to recurring environmental stress through SPL transcription factors. Plant Cell 26, 1792–1807. doi: 10.1105/tpc.114.123851 24769482 PMC4036586

[B37] Stone J. M. Liang X. Nekl E. R. Stiers J. J. (2005). Arabidopsis AtSPL14, a plant-specific SBP-domain transcription factor, participates in plant development and sensitivity to fumonisin B1. Plant J. 41, 744–754. doi: 10.1111/j.1365-313X.2005.02334.x 15703061

[B38] Taguchi-Shiobara F. Yuan Z. Hake S. Jackson D. (2001). The fasciated ear2 gene encodes a leucine-rich repeat receptor-like protein that regulates shoot meristem proliferation in maize. Genes Dev. 15, 2755–2766. doi: 10.1101/gad.208501 11641280 PMC312812

[B39] Tian T. Liu Y. Yan H. You Q. Yi X. Du Z.. (2017). agriGO v2.0: a GO analysis toolkit for the agricultural community 2017 update. Nucleic Acids Res. 45, W122–W129. doi: 10.1093/nar/gkx382 PMC5793732 28472432

[B40] Tilman D. Balzer C. Hill J. Befort B. L. (2011). Global food demand and the sustainable intensification of agriculture. Proc. Natl. Acad. Sci. U.S.A. 108, 20260–20264. doi: 10.1073/pnas.1116437108 22106295 PMC3250154

[B41] Tsuda K. Abraham-Juarez M.-J. Maeno A. Dong Z. Aromdee D. Meeley R.. (2017). KNOTTED1 cofactors, BLH12 and BLH14, regulate internode patterning and vein anastomosis in maize. Plant Cell 29, 1105–1118. doi: 10.1105/tpc.16.00967 28381444 PMC5466031

[B42] Vegetti A. Anton A. M. (1995). Some evolution trends in the inflorescence of poaceae. Flora 190, 225–228. doi: 10.1016/S0367-2530(17)30655-2

[B43] Vollbrecht E. Springer P. S. Goh L. Buckler E. S. Martienssen R. (2005). Architecture of floral branch systems in maize and related grasses. Nature 436, 1119–1126. doi: 10.1038/nature03892 16041362

[B44] Voorend W. Nelissen H. Vanholme R. De Vliegher A. Van Breusegem F. Boerjan W.. (2016). Overexpression of GA20-OXIDASE1 impacts plant height, biomass allocation and saccharification efficiency in maize. Plant Biotechnol. J. 14, 997–1007. doi: 10.1111/pbi.12458 26903034 PMC5019232

[B45] Wang J.-W. Czech B. Weigel D. (2009). miR156-regulated SPL transcription factors define an endogenous flowering pathway in arabidopsis thaliana. Cell 138, 738–749. doi: 10.1016/j.cell.2009.06.014 19703399

[B46] Wang B. Lin Z. Li X. Zhao Y. Zhao B. Wu G.. (2020). Genome-wide selection and genetic improvement during modern maize breeding. Nat. Genet. 52, 565–571. doi: 10.1038/s41588-020-0616-3 32341525

[B47] Wang H. Wang H. (2015). The miR156/SPL module, a regulatory hub and versatile toolbox, gears up crops for enhanced agronomic traits. Mol. Plant 8, 677–688. doi: 10.1016/j.molp.2015.01.008 25617719

[B48] Wang S. Wu K. Yuan Q. Liu X. Liu Z. Lin X.. (2012). Control of grain size, shape and quality by OsSPL16 in rice. Nat. Genet. 44, 950–954. doi: 10.1038/ng.2327 22729225

[B49] Wang C. Yang X. Zhang Y. Shen C. Shi J. Xia C.. (2022). Barley FASCIATED EAR genes determine inflorescence meristem size and yield traits. Crop J. doi: 10.1016/j.cj.2022.10.001

[B50] Winkler R. G. Helentjaris T. (1995). The maize Dwarf3 gene encodes a cytochrome P450-mediated early step in gibberellin biosynthesis. Plant Cell 7, 1307–1317. doi: 10.1105/tpc.7.8.1307 7549486 PMC160953

[B51] Woodward J. B. Abeydeera N. D. Paul D. Phillips K. Rapala-Kozik M. Freeling M.. (2010). A maize thiamine auxotroph is defective in shoot meristem maintenance. Plant Cell 22, 3305–3317. doi: 10.1105/tpc.110.077776 20971897 PMC2990124

[B52] Wu L. Luo Z. Shi Y. Jiang Y. Li R. Miao X.. (2022). A cost-effective tsCUT&Tag method for profiling transcription factor binding landscape. J. Integr. Plant Biol. 64, 2033–2038. doi: 10.1111/jipb.13354 36047457

[B53] Wu G. Park M. Y. Conway S. R. Wang J.-W. Weigel D. Poethig R. S. (2009). The sequential action of miR156 and miR172 regulates developmental timing in arabidopsis. Cell 138, 750–759. doi: 10.1016/j.cell.2009.06.031 19703400 PMC2732587

[B54] Wu G. Poethig R. S. (2006). Temporal regulation of shoot development in arabidopsis thalianaby miR156 and its target SPL3. Development 133, 3539–3547. doi: 10.1242/dev.02521 16914499 PMC1610107

[B55] Xie K. Wu C. Xiong L. (2006). Genomic organization, differential expression, and interaction of SQUAMOSA promoter-Binding-Like transcription factors and microRNA156 in rice. Plant Physiol. 142, 280–293. doi: 10.1104/pp.106.084475 16861571 PMC1557610

[B56] Zhang D. Yuan Z. (2014). Molecular control of grass inflorescence development. Annu. Rev. Plant Biol. 65, 553–578. doi: 10.1146/annurev-arplant-050213-040104 24471834

[B57] Zhu Z. Tan L. Fu Y. Liu F. Cai H. Xie D.. (2013). Genetic control of inflorescence architecture during rice domestication. Nat. Commun. 4, 2200. doi: 10.1038/ncomms3200 23884108 PMC3731664

