# Supplementary material for: Genetic mapping and functional analysis of a classical tassel branch number mutant Tp2 in maize
Source: Front Plant Sci. 2023 Jun 2;14:1183697. doi: 10.3389/fpls.2023.1183697 (PMC10275490; doi:10.3389/fpls.2023.1183697)
Supplement: Supplementary file 2 [file DataSheet_1.pdf]

Supplementary Material

Genetic Mapping and Functional Analysis of a classical tassel branch number Mutant *Tp2* in maize

Juan Li[1†], Xi Wang[1†], Junfeng Wei[1], Xinxin Miao[1], Xiaoyang Shang[1], Lin Li[1,2]*

***Correspondence:**
Lin Li
hzaulilin@mail.hzau.edu.cn

Supplementary Figures

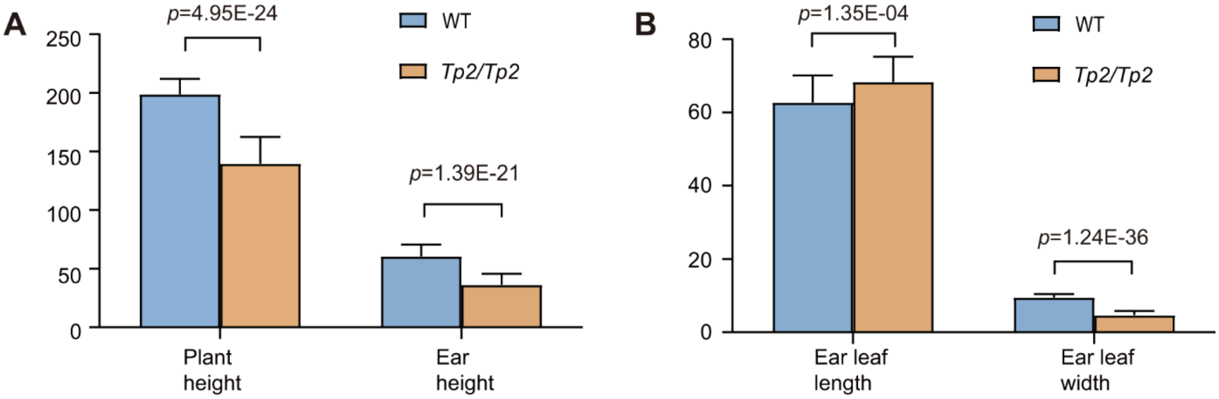

Supplementary Figure 1. The phenotypes of wild-type and mutant in the F_2 segregating population of *Tp2*. (**A-B**) Statistical analysis of plant height and ear height (**A**), ear leaf length and ear leaf width (**B**). The unit is centimeter and the significant difference is examined using a two tailed Student's t-test.

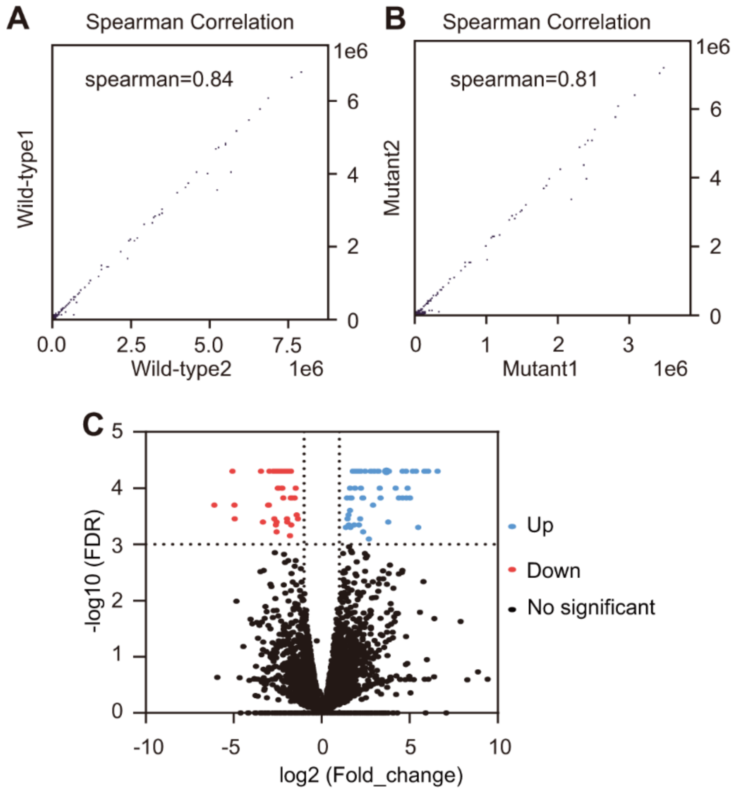

Supplementary Figure 2. The analysis of mRNA-Seq data. (**A-B**) The Spearman's rank correlation coefficient of the two biological replicates from RNA library of homozygous wild-type (**A**) and homozygous mutant (**B**). (**C**) Volcano plots in analyzing differentially expressed genes with mRNA sequencing.

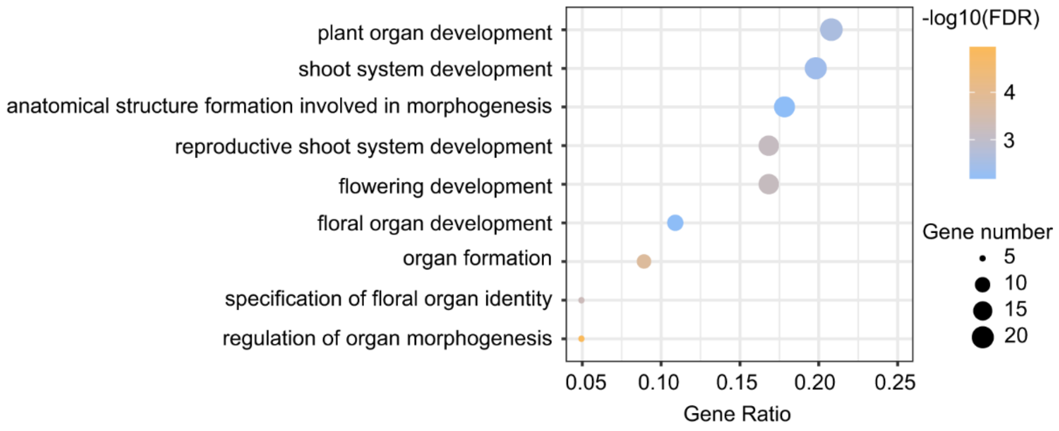

Supplementary Figure 3. GO analysis of differentially expressed genes in tassel from wild type (WT) and *Tp2/Tp2* of two biological replicates.

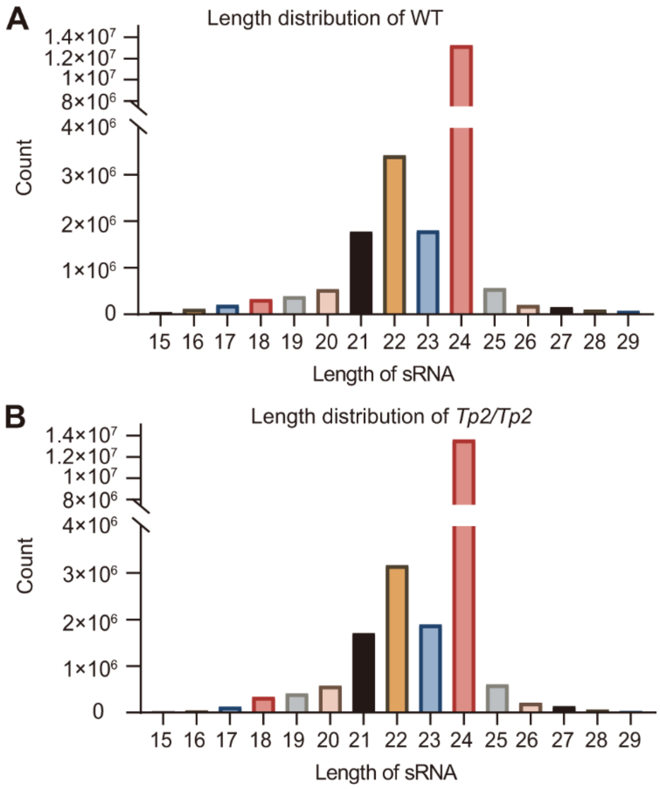

Supplementary Figure 4. The length distribution of sRNA in SAM from wild type (WT) and *Tp2/Tp2* of two biological replicates.

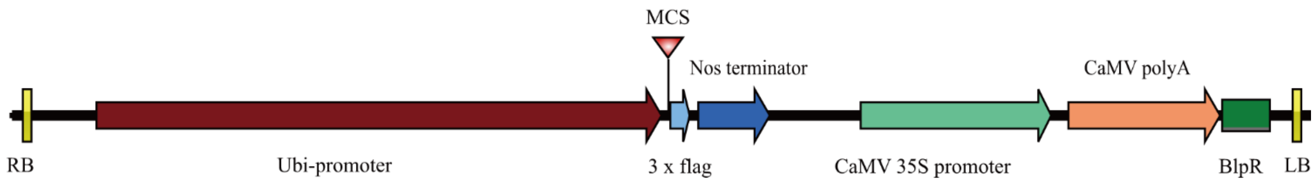

Supplementary Figure 5. The Structure of the overexpression vector.

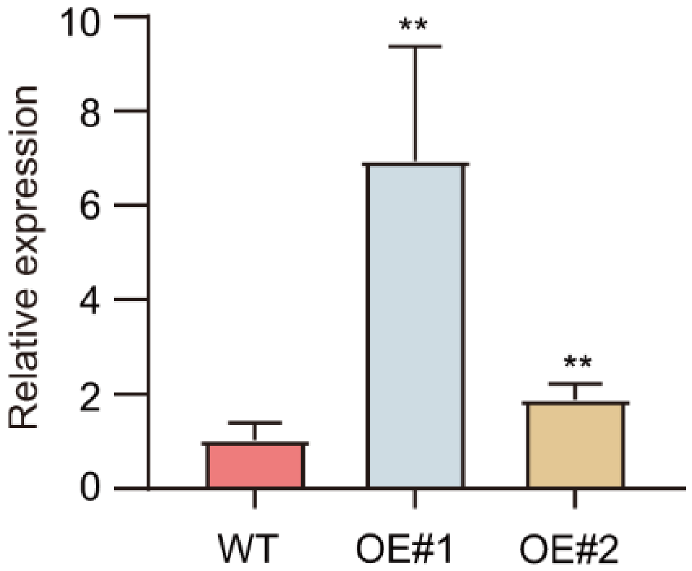

Supplementary Figure 6. Relative expression levels of *zma-miR156h* in OE-plants and their control via qRT-PCR assay, respectively. Double asterisks represent significance difference determined by the Student's t-test at $P < 0.01$.

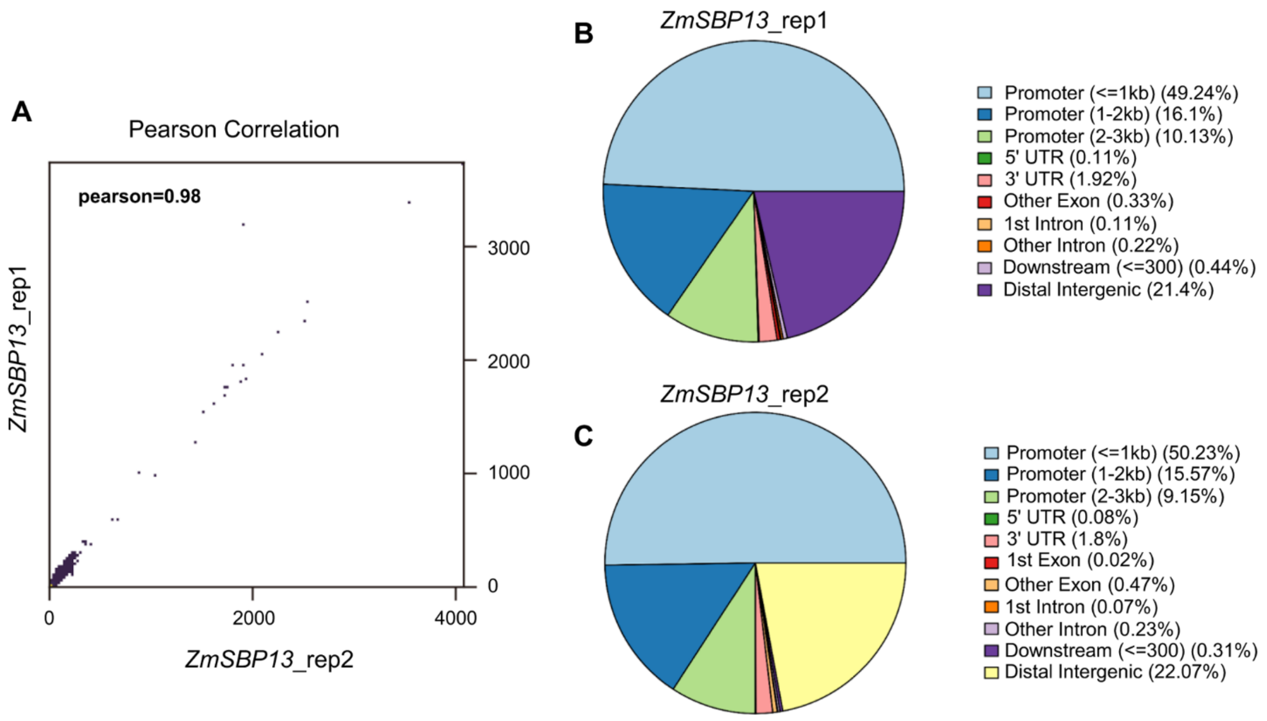

Supplementary Figure 7. The data quality of tsCUT&Tag. (**A**) Scatter plots of *ZmSBP13* tsCUT&Tag data. (**B-C**) Distribution of peaks in genomic regions of the tsCUT&Tag. The Pearson correlations were calculated in deepTools (version 3.5.0).
